# Yield, variability, reliability, and stability of two-dimensional materials based solid-state electronic devices

**DOI:** 10.1038/s41467-020-19053-9

**Published:** 2020-11-10

**Authors:** Mario Lanza, Quentin Smets, Cedric Huyghebaert, Lain-Jong Li

**Affiliations:** 1 Physical Sciences and Engineering Division, King Abdullah University of Science and Technology (KAUST), Thuwal, 23955-6900 Saudi Arabia; 2 IMEC, Kapeldreef 75, 3001 Heverlee (Leuven), Belgium; 3 Department of Electronic Engineering, and Green Technology Research Center, Chang-Gung University, Taoyuan, 333 Taiwan

**Keywords:** Electrical and electronic engineering, Two-dimensional materials

## Abstract

The importance of statistical analyses on 2D materials-based electronic devices and circuits is sometimes overlooked. Here the authors discuss the most pressing integration issues for such devices and emphasize the need for yield, variability, reliability, and stability benchmarking, and outline viable strategies resulting in research papers that are useful for the industry.

Taking advantage of the outstanding properties of two-dimensional (2D) materials to fabricate advanced solid-state electronic devices *beyond* the complementary metal oxide semiconductor technology is an attractive approach that may provide a solution to extend Moore’s law^1,2^. A plethora of studies have reported the fabrication of 2D materials-based electronic devices with excellent performance, such as field effect transistors (FETs) that exhibit current on/off ratios >10^9^ and subthreshold swing (SS) ~62 mV/decade^3^, photodetectors with high form factor and modulation bandwidths for communication beyond 180 Gb/s^4^, and memristors with excellent flexibility, transparency, and thermal stability^5,6^. In order to provide useful technological knowledge, research papers in the field of 2D nano/micro-electronics should satisfy four requirements: (i) all methods used for the synthesis of the materials and the fabrication of the devices must be scalable to wafer level (i.e. mechanical exfoliation of bulk crystals should be avoided), (ii) the morphology and density of non-idealities in the 2D materials used (e.g. thickness fluctuations, lattice distortions) should be clearly specified and statistically demonstrated, (iii) the size of the devices must be small enough to be compatible with the integration density requirements of the target technology (in general, for exploratory studies, lateral sizes <100 nm are recommended), and (iv) information about yield, device-to-device variability, reliability, and stability (including descriptions of the main failure mechanisms for each type of device) must be provided. Within the available literature, still few reports adopted these methods and provide such information.

The year 2019 marked the achievement of important milestones towards the wafer-scale production of 2D materials-based microelectronic devices, which overcame critical difficulties on material synthesis and device patterning. Taiwan Semiconductor Manufacturing Company demonstrated the fabrication of p-type FETs with 6-layer-thick and 40 nm-long WS_2_ channels that exhibit 10^6^ on/off current ratio, SS of ~97 mV/decade, and nearly zero drain-induced barrier lowering^7^. IMEC developed n-type FETs with ~3-layer-thick MoS_2_, with 29 nm channel length and 13 nm contact length, and achieved an on-state current of ~250 μA/μm and an excellent SS = 80 m V/decade using 50 nm SiO_2_ and 4 nm HfO_2_ as back-gate dielectric, respectively^8^. Ref. ^8^ presents a variability study of the SS, threshold voltage (*V*_T_) and contact resistance (*R*_C_) for hundreds of devices, which allowed the authors to design a detailed strategy for future optimizations. Similar studies were carried earlier in academia^9^ but not in such small devices. As it is expected that many other papers in this direction will follow in the next years^1,2,10,11^, here we discuss the status and prospects of the yield, variability, reliability, and stability of solid-state microelectronic devices (mainly FETs and memristors) made of layered 2D materials.

## Yield and device-to-device variability

Device yield is defined as the percentage of devices that work properly, according to specifications and within tolerance windows, among the total number of devices measured, and it is an essential magnitude to evaluate the quality of the fabrication process and the maturity of an integrated device^12^. The device-to-device variability is defined as the deviation of the main chosen parameters and figures of merit of the devices, such as carriers’ mobility, gate oxide leakage current, subthreshold swing, and threshold voltage in FETs, form factor and modulation bandwidths in photodetectors, and switching voltages and state resistances in memristors. A detailed list of parameters and their target values for each device and its applications can be found in the last updated version of the International Roadmap of Devices and Systems (IRDS)^13^. The device-to-device variability is normally evaluated by calculating the coefficient of variance (*C*_V_), which can be calculated as the standard deviation divided by the mean value^14^. Both yield and device-to-device variability are related to the introduction of different types and amounts of defects during the fabrication process of the devices, including material synthesis, aging during storage, transfer process, patterning steps, and deposition of other materials (e.g. contacts). In the case of 2D materials-based devices, the most common intrinsic defects are vacancies, impurities, atomic misalignments, strained bonding, impurities, cracks, wrinkles, and thickness fluctuations in the 2D sheet, while the most common extrinsic defects are related to the changing interaction with the environment resulting in variable adhesion and interaction with the adjacent materials. When the influence of the defects introduced is small, it can modify the characteristics of the devices, resulting in an increase of the device-to-device variability but within an accepted window of operation. However, if the influence of the defects introduced is too large, then the device may fail to perform the actions required, resulting in a decrease of the yield.

**Fig. 1:**
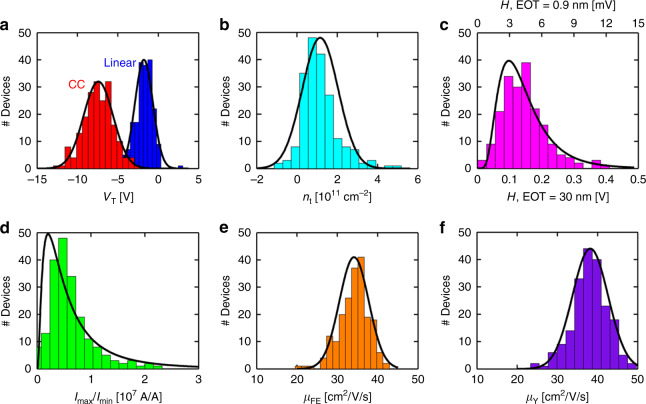
Example of an outstanding variability analysis of multiple parameters of hundreds of MoS_2_ FETs, which furthermore are grown using a scalable method (i.e. CVD). **a** shows the value of *V*_TH_ for both the linear extrapolation (blue) and constant-current (red) methods, **b** shows the value of *n*_t_, **c** the value of the hysteresis, **d** the *I*_MAX_/*I*_MIN_ ratio, and **e**, **f** the values of the mobility extracted from the field-effect approach and the *Y*-function approach (respectively). Reproduced with permission from ref. ^9^ Copyright American Chemical Society, 2017.

Ref. ^9^ fabricated and measured hundreds of back-gate MoS_2_ FETs, spread over an area >1 cm^2^, with channel widths (*W*) of 11.74 ± 0.13 µm, channel lengths (*L*) ranging between 4 µm and 9 µm, and channel thicknesses mainly monolayer with few bilayer islands (≤0.5 μm^2^). The authors reported values of *V*_T_ of −1.78 ± 1.05 V, density of charge traps (*n*_t_) of (1.1 ± 0.9) × 10^11^ cm^−2^, hysteresis (*H*) of 0.14 ± 0.07 V, current max/min ratio (log_10_[*I*_MAX_/*I*_MIN_]) of 6.68 ± 0.40, and carriers’ mobility (*μ*) of 34.2 ± 3.6 cm^2^/V/s (see Fig. 1). This statistical evaluation of the key parameters gives a good view on the potential material performance, and in parallel it highlights the parameters which are the most sensitive to variability and which will require attention in device tuning for stable circuitry. Ref. ^9^ indicated that ultra-low MoS_2_ roughness after fabrication (~0.3 nm), the use of planar Ag/Au contact electrodes, and the use of ultra-clean environment are essential to achieve such low variability. It was also reported that, for the back-gate FETs configuration of those sizes, the presence of bilayer islands in the MoS_2_ channel does not increase the variability of these parameters. In ref. ^8^, 60° twin boundaries were identified as the main type of dislocation present in the MoS_2_ channels grown by chemical vapour deposition (CVD), but the impact of individual dislocations and bilayer islands on electrical performance of individual nanoscale devices could not yet be established. However, the large amount of data collected allowed the authors to discern that the standard deviation of *V*_T_ increases with narrower *W* but not with shorter *L*, suggesting that the Schottky contacts are responsible for *V*_T_ variability. Following up on this statistical approach, ref. ^15^ not only fabricated MoS_2_ FETs at the wafer level (using liquid phase exfoliation) with reasonably stable *μ* but also used the devices to construct logic gates. However, the main drawback of that work is that the size of the devices is too large (*L* ~ 100 µm). Recently, ref. ^16^ reported the fabrication of operational amplifiers using MoS_2_ FETs with channel lengths down to few micrometers (i.e. the smallest FET used has *W* = 5 µm and *L* = 10 µm) and presented transfer characteristics with a low device-to-device variability. Additional effort and focus will be required for high-density electronic circuits made of 2D materials-based FETs with nanoscale dimensions and integrated through state-of-the-art wafer-level processes.

In the field of memristors, the effect of materials defects on the variability is different, as the current does not flow along the 2D material but across it. In such vertical devices, the resistive switching (RS) is a stochastic phenomenon that always takes place at the electrically weakest location of the active area of the device under electrical field. In this context, the presence of cracks in the 2D materials reduce the physical thickness and therefore promote RS at that specific location^17^ (i.e. reduce the switching voltage). Lattice distortions and dopants normally act as trapping sites, facilitating out-of-plane charge transfer and generation of additional defects, which also tend to promote RS and reduce the forming—and switching voltages^18^. On the contrary, wrinkles and polymer residues from the transfer are insulating and increase the out-of-plane resistance, meaning that RS will never take place at those sites^19^; this merely represents a reduction of the effective area of the memristors and it has no remarkable effect in their variability if the samples are relatively clean (i.e. <50 nm^2^ of contaminants per µm^2^). In the field of 2D materials-based memristors, the information available about yield and device-to-device variability is so far scarce. Ref. ^20^ claimed the fabrication of Ag/SnO_*X*_/SnSe memristors with a yield of 100% (out of 80 devices) and presented statistics for the set and reset voltages (*V*_SET_ and *V*_RESET_, respectively). However, the window of operation defining the device yield-pass criteria is expected to be narrower in industrial memristive circuits, and therefore the circuit yield would drop substantially. Refs. ^21,22^ also presented variability information of the switching voltages for few (<10) devices. So far, the most complete report in this direction is the one in ref. ^23^, which analysed hundreds of Au/hexagonal boron nitride (h-BN)/Au memristors and reported yield >98% and device-to-device variability of switching voltages comparable (if not smaller) to that of metal/oxide-based memristors fabricated at industrial facilities^24^, i.e. *C*_V_ of *V*_SET_ ~5.74%. Ref. ^23^ also reported that the variability of the currents in high resistive state (HRS) and low resistive state (LRS) is low enough to ensure 100% state recognition in >1500 cycles measured in 48 devices, even at low LRS currents <500 nA (which are highly desirable to reduce sneak path currents and power consumption). These statistical demonstrations have helped to clarify the real potential of h-BN for memristive technologies, and conducting similar analyses in memristors made of other 2D materials is highly recommended.

### Reliability and stability

In the field of 2D materials-based microelectronic devices, reliability is defined as the time that one electronic device can continuously operate in a predefined operation window. This is determined by the device degradation and failure due to the application of stresses during operation, which can be electrical, mechanical, thermal, chemical, and magnetic^25^. Stability refers to the degradation of the properties of the devices with time, unrelated to operational stresses, but instead by contamination produced by e.g. the relative humidity of the environment and/or atomic diffusion. Note that, under this definition, concepts like thermal stability are included in the term reliability, as it only applies to the devices under operation. Both reliability and stability could be understood as a time-dependent variability, and therefore the failure strongly depends on the window (i.e. criteria) set for each device, circuit, and/or application.

The failure of a microelectronic circuit can have its origin within a device (i.e. front-end of line), at the interconnections between them (i.e. back-end of line), or at the materials used during encapsulation (i.e. packaging)^25^. While studying packaging issues may not be responsibility of academics, dealing with front-end and back-end failure mechanisms should be a priority; however, so far very few authors made remarkable efforts in this direction. Ref. ^26^ analysed the stress-induced leakage current and time-dependent dielectric breakdown in h-BN dielectric stacks using both conductive atomic force microscopy and device-level stresses and concluded that the failure is triggered by the generation of boron vacancies. Refs. ^27,28^ reported that charge trapping and de-trapping in the dielectrics of FETs with MoS_2_ channels increases the hysteresis and negative bias temperature instability and that these problems can be reduced using 2D layered or crystalline self-passivated materials (such as h-BN and CaF_2_, respectively). Ref. ^29^ reported that trilayer graphene barrier stacks prevent Cu diffusion much better than ~3 nm TaN films (which is the industrial standard) and that it could represent a very useful strategy to reduce *R*_C_ and improve the reliability of back-end of line interconnections in microelectronic circuits. Regarding stability, some 2D materials like black phosphorous and silicene (among others) have shown rapid degradation when exposed to air environment, which emphasizes the need to monitor the electrical properties of the devices over storage time. In ref. ^30^, the authors observed that black phosphorous FETs could keep their performance for >17 months using a 25-nm-thick Al_2_O_3_ capping layer, and this strategy also remarkably increased the stability of silicene-based FETs^31^.

In the field of memristors, the concept of reliability is linked to cycle-to-cycle variability of the electrical characteristics. Some figures of merit, such as the endurance and the retention plots can give some idea about how the values of the resistance in HRS and LRS evolve with the number of cycles and time (respectively)^17^, but other parameters such as the switching time and energy have not been analysed depending on the number of cycles. Moreover, the stability of 2D materials-based memristors has been only partially analysed^32^, and more studies in this direction are necessary.

It should be highlighted that the degradation of 2D materials-based microelectronic devices is considerably connected with energy dissipation during device operation. In FETs, non-equilibrium charge carriers flowing in-plane at the channel region can undergo energy relaxation either with the lattice of the 2D material or with the adjacent layers (i.e. substrate, gate dielectric, electrodes), which makes necessary investigating multiple physical phenomena, such as thermal radiation from hot electrons, electron–electron scattering, scattering with optical phonons in the substrate and/or dielectric, thermal decoupling of hot electrons from acoustic phonons, electron–hole recombination, and Peltier effect^33^. These phenomena are strongly related to the thermal conductance of the 2D materials, and while it has been widely demonstrated that their high in-plane thermal conductivities (provided by covalent bonds) enhance the performance of the FETs^34^, inefficient heat transfer out of plane (due to van der Waals gap) and towards adjacent materials (due to disordered bonding) may remarkably decrease the reliability of the devices^35^. In memristors, the out-of-plane current requires the formation of local defects in the lattice of the 2D layered dielectric, and the chemical stability of the materials and energy for defect formation (either intrinsic vacancies or metallic ion penetration) as the electrical stress proceeds play a more important role^17,36^. For both types of devices, additional investigations linking energy dissipation phenomena with device reliability and lifetime are highly necessary.

## Discussion

Fabricating nanoscale devices made of 2D materials to wafer level using scalable methods while achieving excellent performance is a significant challenge even for the most advanced companies. The performance metrics reported for devices with synthetic materials are still severely degraded compared to devices with mechanical exfoliation. As an example, in 2010 an h-BN encapsulated graphene FET was fabricated via mechanical exfoliation, and a carriers’ mobility of 60,000 cm^2^/V/s was observed^37^; the same experiment was repeated 8 years later using CVD-grown graphene and h-BN^38^, and the average mobility observed was only 2500 cm^2^/V/s. While research on mechanically exfoliated 2D materials may still be relevant as reference to evaluate materials performance, there is an urgent need for statistical investigations dealing with integration issues of 2D materials-based microelectronic devices and circuits, the up-scalability of the methods, and targeting industry-standard performance metrics. In this regard, it is worth noting that the parameters and figures of merit of each device depend on the application. As an example, the FETs used in logic gates need to show performances and fit reliability criteria different to those used (for example) to control the current across a memristor. Similarly, memristors used as non-volatile memory and memristors used as electronic synapses need to exhibit different performances. For this reason, it may not be straightforward to provide exact values herein; a complete list of parameters and their target values for different applications can be found in last updated edition of the IRDS^13^. While extended guidelines on how to evaluate the yield, reliability, variability, and stability of transistors^39,40^ and memristors^17,41^ can be found in the literature, some general criteria include: (i) fabricating and characterizing multiple devices, (ii) presenting statistical information of all the parameters and figures of merit determining the reliability of the devices, and (iii) analysing and discussing the failure mechanisms based on experimental measurements with nanometric (if not atomic) resolution, avoiding to only rely on schematics based on intuition. The importance of statistical analyses on 2D devices needs to be emphasized on a broader level in order to enable a technological shift, particularly because critical integration issues risk to be overlooked in academic publications.

## References

[1] Li M-Y (2019). How 2D semiconductors could extend Moore’s law. Nature.

[2] Akinwande D (2019). Graphene and two-dimensional materials for silicon technology. Nature.

[3] Xu J (2017). A two-dimensional semiconductor transistor with boosted gate control and sensing ability. Sci. Adv..

[4] Schall, D. et al. Record high bandwidth integrated graphene photodetectors for communication beyond 180 Gb/s. In *2018 Optical Fiber Communication Conference and Exposition* 1–3 (IEEE, 2018).

[5] Wang M (2018). Robust memristors based on layered two-dimensional materials. Nat. Electron..

[6] Yao J (2012). Highly transparent nonvolatile resistive memory devices from silicon oxide and graphene. Nat. Commun..

[7] Cheng, C.-C. et al. First demonstration of 40-nm channel length top-gate WS2 pFET using channel area-selective CVD growth directly on SiOX/Si substrate. In *Symposium on Very Large Scale Integration Technology* T244–T245 (IEEE, 2019).

[8] Smets, Q. et al. Ultra-scaled MOCVD MoS_2_ MOSFETs with 42 nm contact pitch and 250 µA/µm drain current. In *2019 International Electron Devices Meeting*. 23.2.1–23.2.4 (IEEE, 2019).

[9] Smithe KKH (2017). Low variability in synthetic monolayer MoS_2_ devices. ACS Nano.

[10] Neumaier D (2019). Integrating graphene into semiconductor fabrication lines. Nat. Mater..

[11] Lin L (2019). Synthesis challenges for graphene industry. Nat. Mater..

[12] El-Kareh, B. et al. Yield management in microelectronic manufacturing. In *Proc. 45th Electronic Components and Technology Conference*. 58–63 (IEEE, 1995).

[13] 2018 International Roadmap of Devices and Systems (IRDS). https://irds.ieee.org/ (2018). The industrial targets related to transistors can found in the section “More Moore”. The information related to the use of memristors as non-volatile electronic memory, as well as many other devices, can be found in the section “Beyond CMOS”.

[14] Adam GC (2017). 3-D memristor crossbars for analog and neuromorphic computing applications. IEEE Trans. Electron. Devices.

[15] Lin Z (2018). Solution-processable 2D semiconductors for high- performance large-area electronics. Nature.

[16] Polyushkin DK (2020). Analogue two-dimensional semiconductor electronics. Nat. Electron..

[17] Lanza, M. et al. Recommended methods to study resistive switching devices. *Adv. Electron. Mater*. **5**, 1800143 (2019).

[18] Shi Y (2018). Electronic synapses made of layered two-dimensional materials. Nat. Electron..

[19] Pan C (2017). Model for multi-filamentary conduction in graphene/hexagonal-boron-nitride/graphene based resistive switching devices. 2D Mater..

[20] Guo J (2020). Highly reliable low-voltage memristive switching and artificial synapse enabled by van der Waals integration. Matter.

[21] Pan C (2017). Coexistence of grain-boundaries-assisted bipolar and threshold resistive switching in multilayer hexagonal boron nitride. Adv. Funct. Mater..

[22] Zhuang P (2020). Nonpolar resistive switching of multilayer-hBN-based memories. Adv. Electron. Mater..

[23] Chen S (2020). Wafer-scale integration of 2D materials in high-density memristive crossbar arrays for artificial neural networks. Nat. Electron..

[24] Fantini, A. et al. Intrinsic switching variability in HfO_2_ RRAM in *2013 5th IEEE International Memory Workshop* 30–33 (IEEE, 2013).

[25] Raghavan, N. et al. in *Reliability Characterisation of Electrical and Electronic Systems* (ed. Swingler, J.) 143–168 (Elsevier, 2015).

[26] Ranjan A (2019). Boron vacancies causing breakdown in 2D layered hexagonal boron nitride dielectrics. IEEE Electron Dev. Lett..

[27] Illarionov YY (2016). The role of charge trapping in MoS_2_/SiO_2_ and MoS2/hBN field-effect transistors. 2D Mater..

[28] Illarionov YY (2019). Ultrathin calcium fluoride insulators for two-dimensional field-effect transistors. Nat. Electron..

[29] Li L (2015). Vertical and lateral copper transport through graphene layers. ACS Nano.

[30] Illarionov YY (2017). Highly-stable black phosphorus field-effect transistors with low density of oxide traps. npj 2D Mater. Appl..

[31] Tao L (2015). Silicene field-effect transistors operating at room temperature. Nat. Nanotechnol..

[32] Yang PK (2013). Fully transparent resistive memory employing graphene electrodes for eliminating undesired surface effects. Proc. IEEE.

[33] Ong Z-Y, Bae M-H (2019). Energy dissipation in van der Waals two-dimensional devices. 2D Mater..

[34] Song H (2018). Two-dimensional materials for thermal management applications. Joule.

[35] Parto, K., Pal, A., Xie, S., Cao, W. & Banerjee, K. Interfacial thermal conductivity of 2D layered materials: an atomistic approach. In *2018 International Electron Devices Meeting* 24.1.1–24.1.4 (IEEE, 2018).

[36] Zobelli A, Ewels CP, Gloter A, Seifert G (2007). Vacancy migration in hexagonal boron nitride. Phys. Rev. B.

[37] Dean CR (2010). Boron nitride substrates for high-quality graphene electronics. Nat. Nanotechnol..

[38] Pandey H (2018). All CVD boron nitride encapsulated graphene FETs with CMOS compatible metal edge contacts. IEEE Trans. Electron. Devices.

[39] Green ML, Gusev EP, Degraeve R, Garfunkel EL (2001). Ultrathin (SiO_2_ and Si–O–N gate dielectric layers for silicon microelectronics: understanding the processing, structure, and physical and electrical limits. J. Appl. Phys..

[40] Hicks J (2008). 45nm transistor reliability. Intel. Technol. J..

[41] Burr G (2017). Neuromorphic computing using non-volatile memory. Adv. Phys. X.

